# Reduced peripheral serotonin levels in women with multiple sclerosis: associations with underweight status, treatment duration, and use of interferon beta 1a

**DOI:** 10.3389/fncel.2026.1752975

**Published:** 2026-03-11

**Authors:** Gilberto Pérez-Sánchez, León Arturo Aguilar-Gómez, Giselle Berenice Vela-Sancho, Salomón Jacinto-Gutiérrez, Enrique Becerril-Villanueva, Samantha Alvarez-Herrera, Luis Vallejo-Castillo, María Paulina Reyes-Mata, Mario Alberto Mireles-Ramírez, José de Jesús Guerrero-García, Daniel Ortuño-Sahagún, Lenin Pavón

**Affiliations:** 1 Laboratorio de Psicoinmunología, Instituto Nacional de Psiquiatría Ramón de la Fuente Muñiz, Ciudad de México, Mexico; 2 Laboratorio de Neuroinmunobiología Molecular, Instituto de Neurociencias Translacionales, Centro Universitario de Ciencias de la Salud, Universidad de Guadalajara, Guadalajara, Jalisco, Mexico; 3 Unidad de Desarrollo e Investigación en Bioterapéuticos (UDIBI), Escuela Nacional de Ciencias Biológicas, Instituto Politécnico Nacional, Mexico City, Mexico; 4 Departamento de Disciplinas filosófico, Metodológicas e Instrumentales, CUCS, Universidad de Guadalajara, Guadalajara, Mexico; 5 Unidad Médica de Alta Especialidad (UMAE), Hospital de Especialidades (HE), Centro Médico Nacional de Occidente (CMNO), Instituto Mexicano del Seguro Social (IMSS), Guadalajara, Mexico; 6 Banco Central de Sangre, Unidad Médica de Alta Especialidad (UMAE), Hospital de Especialidades (HE), Centro Médico Nacional de Occidente (CMNO), Instituto Mexicano de Seguro Social (IMSS), Guadalajara, Jalisco, Mexico; 7 Departamento de Farmacobiología, Centro Universitario de Ciencias Exactas e Ingenierías (CUCEI), Universidad de Guadalajara, Guadalajara, Jalisco, Mexico

**Keywords:** 5-HIAA, interferon beta, treatment duration, underweight, multiple sclerosis, RRMS, serotonin, tryptophan

## Abstract

Multiple sclerosis (MS) is a chronic autoimmune-mediated demyelinating disease of the CNS, characterized by neuroinflammatory, axonal degeneration, and pronounced sexual dimorphism. Experimental data implicate dysregulated 5-HT levels in MS. However, the effects of clinical parameters and disease-modifying-therapies (DMTs) on peripheral 5-HT concentrations remain underexplored. This study aimed to quantify peripheral levels of tryptophan (Trp), 5-HT, and 5-hydroxyindoleacetic acid (5-HIAA) in patients with relapsing–remitting MS (RRMS) and to assess the effects of BMI, DMT duration, and specific DMT regimens. In this cross-sectional analysis, 226 participants were enrolled and stratified into four groups: healthy men (HM; *n* = 29), healthy women (HW; *n* = 84), men with RRMS (MMS; *n* = 29), and women with RRMS (WMS; *n* = 84). Serum concentrations of Trp, 5-HT, and 5-HIAA were measured using reverse-phase high-performance liquid chromatography (HPLC) with fluorescence detection. Nonparametric statistical tests were applied. Peripheral 5-HT levels were significantly reduced in underweight WMS (BMI < 18 kg/m^2^; *p* < 0.05), WMS with DMT duration over 4 years (*p* < 0.01), and WMS receiving interferon beta-1a (*p* < 0.01) compared to HW. No significant intergroup differences in Trp or 5-HIAA were observed across all stratifications. These findings reveal a novel association between reduced peripheral 5-HT and specific clinical-therapeutic factors in WMS, extending recent MS research on sex-specific vulnerabilities, serotonergic dysregulation in neuroinflammation, and psychiatric comorbidity. By highlighting the influence of low BMI, prolonged DMT exposure, and interferon beta-1a on 5-HT homeostasis, this study underscores the need for multidisciplinary management integrating neurological and psychiatric care in WMS and suggests avenues for precision interventions targeting serotonergic pathways to reduce disease burden.

## 1 Introduction

Multiple sclerosis (MS) is a chronic autoimmune-mediated demyelinating disease affecting the central nervous system (CNS), characterized by neuroinflammation, demyelination, gliosis, and neuronal loss (Salihu et al., 2021). Clinical manifestations include visual impairment, sensory disturbances such as numbness and tingling, focal weakness, bladder or bowel dysfunction, and cognitive deficits, with symptoms correlating to lesion location and often presenting as relapses in young adults (Noyes and Weinstock-Guttman, 2013). MS exhibits marked sexual dimorphism, with females affected three times more frequently than males (Gilli et al., 2020; Tafti et al., 2025). However, males typically experience more severe progression and greater neurological damage (Gilli et al., 2020). Immune cells perivascular infiltration contributes to myelin degradation, exacerbating pathology (Adams et al., 1989).

MS prevalence and incidence are rising globally, with over 2.8 million cases worldwide (S.F.M.S.E.T. University of California et al., 2016). This trend reflects demographic changes and improved diagnostics, rather than increased individual risk (S.F.M.S.E.T. University of California et al., 2016; Portaccio et al., 2024). In Europe, the average societal cost per MS patient was €40,303 in 2015 and varies by country. Productivity loss accounted for 39% of total costs, drug expenses for 21%, and informal caregiving for 15%. Societal costs rise with disease severity: mild MS costs $22,719 annually, and severe cases reach $64,853 (2011 values). Costs also differ by type: relapsing–remitting MS costs €31,007 per patient annually, while secondary progressive MS costs €58,475 (2021 values) (Wang et al., 2025).

Disease-modifying therapies (DMTs) are standard for MS management (Simoens, 2022), including anti-B-cell antibodies, sphingosine-1-phosphate modulators, fumarates, immunomodulators like glatiramer acetate, and immunosuppressants such as natalizumab, mitoxantrone, and corticosteroids. Glatiramer acetate promotes a shift from proinflammatory Th1 to regulatory Th2 responses, reducing proinflammatory cytokines (IL-2, IL-12) while enhancing anti-inflammatory cytokines (IL-1, IL-4, IL-10), and is linked to fewer depressive symptoms in RRMS (Jakimovski et al., 2024; Ziemssen et al., 2016). In contrast, interferon beta-1a (IFN-β1a), used for relapsing MS, modulates cytokine and matrix metalloproteinase expression and restores T-cell suppression (Liu et al., 2021). but is associated with depression potentially via serotonin (5-HT) reduction (Bonaccorso et al., 2009).

Dysregulated tryptophan (Trp) metabolism in MS diverts Trp from 5-HT synthesis toward the kynurenine pathways, driven by inflammation, thereby lowering 5-HT levels and influencing neuroinflammation, disease progression, and psychiatric comorbidities (Gaetani et al., 2020; Nourbakhsh et al., 2018). Sex differences in Trp metabolism (Pais et al., 2023) may contribute to dimorphic MS outcomes. Despite these insights, the impact of clinical variables like body mass index (BMI), DMT duration, and specific regimens on peripheral Trp, 5-HT, and 5-hydroxyindoleacetic acid (5-HIAA) levels remains underexplored, particularly in sex-stratified cohorts. This novel study quantifies these metabolites in RRMS patients and evaluates the influences of BMI, treatment duration, and DMTs (glatiramer acetate, IFN-β, fingolimod, dimethyl fumarate, rituximab, natalizumab), providing evidence-based rationale for targeted serotonergic interventions and integrated care to address MS burden.

## 2 Materials and methods

### 2.1 Patients and healthy control subjects

A total of 113 patients with relapsing–remitting multiple sclerosis (RRMS) and 113 healthy controls participated in this cross-sectional analysis. Table 1 presents their clinical and demographic profiles. The study was conducted in the Neurology Department of the UMAE Hospital de Especialidades at the Centro Médico Nacional de Occidente, Instituto Mexicano del Seguro Social, Jalisco, Mexico. Inclusion criteria were age 20–60 years and diagnosis according to the 2018 McDonald criteria (Thompson et al., 2018). Participants received IFN-β1a, fingolimod, natalizumab, or rituximab for at least 3 months, or had no treatment for at least 2 years. Clinical disability was measured using the Expanded Disability Status Scale (EDSS) (Kurtzke, 1983). Disease subtypes were classified according to the Lublin and Reingold classification (S.F.M.S.E.T. University of California et al., 2016; Lublin et al., 2014).

**Table 1:** Demographic and clinical characteristics of participants.

Clinical and demographic variables	Healthy men (HM) *n* = 29	Men with MS (MMS) *n* = 29	Healthy women (HW) *n* = 84	Women with MS (WMS) *n* = 84
Age (years)	39.1 ± 10.4	34.5 ± 8.1	35.5 ± 8.5	40.7 ± 11.9
BMI	24.1 ± 4.1	25.2 ± 4.0	26.3 ± 3.9	22.7 ± 5.1
Weight status of participants	Underweight: *n* = 0 Normal weight: *n* = 6 Overweight: *n* = 15 Obesity *n* = 8	Underweight: *n* = 0 Normal weight: *n* = 12 Overweight: *n* = 8 Obesity *n* = 5 Without data *n* = 4	Underweight: *n* = 0 Normal weight: *n* = 15 Overweight: *n* = 42 Obesity *n* = 27	Underweight: *n* = 12 Normal weight: *n* = 3 Overweight: *n* = 39 Obesity *n* = 24 Without data *n* = 6
Length of treatment (years)		≤ 3 years: *n* = 18 ≥3 years: *n* = 11		≤3 years: *n* = 50 ≥3 years: *n* = 34
Pharmacological treatments		U: *n* = 3 RTX: *n* = 9 NTZ: *n* = 3 IFNB: *n* = 5 GA: *n* = 6 DMF: *n* = 2 FNG: *n* = 1 Other: 0		U: *n* = 8 RTX: *n* = 17 NTZ: *n* = 7 IFNB: *n* = 20 GA: *n* = 10 DMF: *n* = 9 FNG: *n* = 12 Other: 1
EDSS score		2.1 ± 0.8		2.4 ± 1.7
Trp, pmol/mL	13,015.0 ± 6,293.0	12,087 ± 1,915.8	13,320.0 ± 3,959.0	11,766.1 ± 2,773.1
5-HT, pmol/mL	305.6 ± 170.1	219.8 ± 64.1	256.2 ± 86.6	258.0 ± 141.6
5-HIAA, pmol/mL	834 ± 415.7	742.9 ± 119.5	851.7 ± 298.3	755.5 ± 245.3

BMI, Body mass index; EDSS, Expanded Disability Status Scale; Trp, tryptophan; 5-HT, serotonin; 5-HIAA, 5-hydroxyindoleacetic acid; U, unmedicated; RTX, rituximab; NTZ, natalizumab; IFNB, interferon betta; GA, glatiramer acetate; DMF, dimetilfumarate; FNG, fingolimod.

A clinical relapse was defined as an acute neurological decline. Remission was defined as partial or full recovery without relapses in the 3 months before enrollment. Exclusion criteria: included secondary or primary progressive MS, corticosteroid treatment within 3 months, history of autoimmune or inflammatory disease, or other chronic CNS diseases. All patients provided written informed consent.

The control group consisted of 113 healthy subjects (84 females, 29 males), matched for age and sex and recruited from the Hospital Central Blood Bank at CMNO-IMSS. To minimize circadian rhythm bias, samples from RRMS patients and controls were collected at the same time of day; control samples were obtained on separate days but at the same hour. The study complied with the 2024 Declaration of Helsinki and received approval from the IMSS (R-2022-785-045) and CUCS (CI-01623) Ethics Committees in Mexico. All participants provided written informed consent.

### 2.2 Collection of blood samples

Blood was collected using a vacuum system into tubes designed for serum. Samples were centrifuged at 2,500 rpm for 10 min at 4 °C. The serum was aliquoted in Eppendorf tubes and stored at −80 °C until use.

### 2.3 Extraction and quantification of tryptophan (Trp), serotonin (5-HT), and 5-Hydroxyindoleacetic acid (5-HIAA)

Trp, 5-HT, and 5-HIAA were measured in 200 μL of serum. Equal volumes of extraction buffer (5% ascorbic acid, 2.5 mM EDTA, 2.5 mM L-cysteine) were added. Then, protein was removed by precipitation with 100 μL of 2.4 M perchloric acid. Samples were incubated at 20 °C for 20 min, then centrifuged at 12,000 rpm for 20 min at 4 °C. The supernatant containing Trp, 5-HT, and 5-HIAA was collected.

To enrich the analytes, supernatants were processed using solid-phase extraction (SPE) columns (Strata C18-E, 55 μm, 70 Å, 100 mg/1 mL; Phenomenex). Quantification of 5-HT and TRP was performed by reversed-phase high-performance liquid chromatography (RP-HPLC). This used a PU-2089 plus pump, an AS-2057 plus autosampler, and an X-LC 3120FP fluorescence detector (Jasco, Inc.). All instruments were controlled with ChromNav software (Jasco, Inc.).

Chromatographic separation was performed on a CAPCELL PACK MGII C18 column (300 Å, 5 μm, 4.6 × 250 mm; SHISEIDO) maintained at 30 °C. The column was first equilibrated with mobile phase A (0.1% trifluoroacetic acid in 2% methanol). A linear gradient was run from minute 3 to minute 43, reaching 10% mobile phase B (0.1% trifluoroacetic acid in acetonitrile). The flow rate was 0.8 mL/min.

Fluorescence detection parameters were: gain 100, attenuation 32, and response time 20 s. Excitation and emission wavelengths were 280 nm and 315 nm, respectively. Sample injection volumes ranged from 10 to 50 μL. Calibration curves were generated for TRP, 5-HT, and 5-HIAA. This ensures accurate quantification across the expected concentration range.

### 2.4 Statistical analysis

All statistical analyses were performed using GraphPad Prism version 10.0.3 (San Diego, CA, USA). The Shapiro–Wilk test was used to assess normality for age, BMI, EDSS, and serum levels of Trp, 5-HT, and 5-HIAA in healthy men (HM), men with MS (MMS), healthy women (HW), and women with MS (WMS). Based on these results, groups were compared using the Kruskal–Wallis test and Dunn’s *post hoc* test. Because the comparison between men and women showed no differences, HM were compared with MMS, and HW were compared with WMS. To analyze the effect of BMI, treatment duration, and treatment type, these groups were stratified by BMI category (<18 underweight, 18–24 normal, 25–30 overweight, >30 obese); the BMI categories of WMS were compared with the normal BMI group of HW; treatment duration (0.1–3.9 years, ≥4 years), and DMT regimen (rituximab, natalizumab, IFN-beta1a, glatiramer acetate, dimethyl fumarate, fingolimod) were compared with the HW group. Statistical significance was defined as *p* < 0.05.

## 3 Results

### 3.1 Demographic and clinical characteristics of participants

Table 1 presents the demographic and clinical characteristics of the participants. There were no statistically significant differences in age, BMI, or EDSS among the MS patient groups. No differences were found in Trp, 5-HT, or 5-HIAA levels. The population’s mean age of the participants was 37 years, and the mean BMI was 24.5. Approximately 60% of patients had less than 3 years of treatment. Overweight status was observed in 60% of men and 80% of women. MS patients had EDSS scores of 2.0–2.5, indicating minimal to mild disability, primarily based on Functional Systems findings. At this level, patients remain ambulatory and can walk at least ≥500 m without aid or rest. EDSS scores are determined by neurological impairments in the Functional Systems (Pyramidal, Cerebellar, Brainstem, Sensory, Bowel/Bladder, Visual, Cerebral) (Kurtzke, 1983).

### 3.2 Influence of BMI status on tryptophan, serotonin, and 5-HIAA levels in multiple sclerosis patients

The detection of TRP, 5-HT, and 5-HIAA in MS patients, stratified by Body Mass Index (BMI), reveals a significant association primarily related to central serotonergic activity, as reflected in CSF metabolites (Markianos et al., 2013). Statistical analysis of our serum samples from MS patients (Table 2) shows significant differences, H(4) = 12.46, *p* < 0.0142. WMS patients with a BMI < 18 have significantly lower serotonin levels (pmol/mL) compared to their respective controls (WMS low BMI 164.2 ± 117.8 vs. HW normal BMI 305.6 ± 170.1; *p* < 0.05). This group also had the lowest TRP levels. In all cases, MMS and WMS patients had lower 5-HIAA levels than their controls.

**Table 2:** Serum levels of Trp, 5-HT, and 5-HIAA in populations stratified by BMI.

BMI status	Healthy men (HM) *n* = 29	Serum levels of neurotransmitters (mean ± SD)	Men with MS (MMS) *n* = 29	Serum levels of neurotransmitters (mean ± SD)	Healthy women (HW) *n* = 84	Serum levels of neurotransmitters (mean ± SD)	Women with MS (WMS) *n* = 84	Serum levels of neurotransmitters (mean ± SD)
BMI < 18 (low weight)	*n* = 0		*n* = 0		*n* = 0		*n* = 12	Trp = 10,394.0 ± 2,782.2 5-HT = 164.2 ± 117.8*^a^ 5-HIAA = 515.5 ± 173.0
BMI 18–24.9 (normal weight)	*n* = 6	Trp = 14,001.0 ± 3,907.0 5-HT = 292.6 ± 111.1 5-HIAA = 835.7 ± 328.9	*n* = 12	Trp = 12,940 ± 1,495.0 5-HT = 276.1 ± 106.4 5-HIAA = 724.8 ± 127.8	*n* = 15	Trp = 12,846.0 ± 2,368.0 5-HT = 305.6 ± 170.1*^a^ 5-HIAA = 778.4 ± 221.4	*n* = 3	Trp = 13,624.0 ± 2,329.0 5-HT = 334.5 ± 121.2 5-HIAA = 724.2 ± 215.3
BMI 25–30 (overweight)	*n* = 15	Trp = 14,809.0 ± 3,365.0 5-HT = 263.1 ± 85.1 5-HIAA = 844.6 ± 225.3	*n* = 8	Trp = 13,158.0 ± 2,821.0 5-HT = 236.1 ± 2,821.0 5-HIAA = 236.1 ± 121.4	*n* = 42	Trp = 14,726.3 ± 1,245.9 5-HT = 266.3 ± 169.4 5-HIAA = 669.2 ± 215.3	*n* = 39	Trp = 14,135.0 ± 1,693.0 5-HT = 241.3 ± 149.3 5-HIAA = 787.5 ± 229.0
BMI > 30 (obesity)	*n* = 8	Trp = 12,187.0 ± 4,187.0 5-HT = 231.8 ± 68.2 5-HIAA = 864.4 ± 331.1	*n* = 5	Trp = 11,107.0 ± 1,856.3 5-HT = 299.6 ± 97.1 5-HIAA = 649.2 ± 128.5	*n* = 27	Trp = 14,973.0 ± 1,968.0 5-HT = 241.5 ± 157.1 5-HIAA = 627.4 ± 161.9	*n* = 24	Trp = 15,064.0 ± 3,996.0 5-HT = 272.2 ± 141.4 5-HIAA = 739.0 ± 263.0
			*n* = 4	patients without BMI data			*n* = 6	patients without BMI data

BMI, Body mass index; Trp, tryptophan; 5-HT, serotonin; 5-HIAA, 5-hydroxyindoleacetic acid; SD, standard deviation. Similar letters indicate the groups being compared. *= *p* < 0.05.

### 3.3 Influence of treatment duration on tryptophan, serotonin, and 5-HIAA levels in multiple sclerosis patients

The effect of Disease-Modifying Therapy (DMT) duration on TRP, 5-HT, and 5-HIAA levels in the serum of MS patients remains poorly defined. However, studies indicate a link between treatment duration and neuroimmune pathways, particularly in immunological regulation and CNS metabolites (Ricci et al., 2025). Statistical analysis of our serum samples from MS patients, shown in Table 3; it shows significant differences (H(2) = 9.74, *p* < 0.0077). WMS patients with a treatment duration of more than 4 years have significantly lower serotonin levels (pmol/mL) compared to their respective controls (WMS > 4Y 210.0 ± 146.0 vs. HWWT 305.6 ± 170.1; *p* < 0.01).

**Table 3:** Serum levels of Trp, 5-HT, and 5-HIAA in populations stratified by treatment duration.

Treatment duration (years)	Healthy men (HM) *n* = 29	Serum levels of neurotransmitters (mean ± SD)	Men with MS (MMS) *n* = 29	Serum levels of neurotransmitters (mean ± SD)	Healthy women (HW) *n* = 84	Serum levels of neurotransmitters (mean ± SD)	Women with MS (WMS) *n* = 84	Serum levels of neurotransmitters (mean ± SD)
	*n* = 29	Trp = 13,320.0 ± 3,959.0 5-HT = 256.2 ± 86.6 5-HIAA = 851.7 ± 298.3			*n* = 84	Trp = 12,846.0 ± 6,449.0 5-HT = 306.0 ± 170.0**^a^ 5-HIAA = 834.0 ± 416.0		
0.1–3.9			*n* = 18	Trp = 12,031 ± 2,321.0 5-HT = 274.2 ± 118.9 5-HIAA = 709.2 ± 126.1			*n* = 50	Trp = 12,394.0 ± 2,776.0 5-HT = 277.0 ± 154.0 5-HIAA = 719.2 ± 178.9
>4			*n* = 11	Trp = 13,265.0 ± 3,716.0 5-HT = 267.7 ± 105.7 5-HIAA = 674.9 ± 268.5			*n* = 34	Trp = 14,327.0 ± 3,662.0 5-HT = 210.0 ± 146.0**^a^ 5-HIAA = 759.8 ± 140.4

Trp, tryptophan; 5-HT, serotonin; 5-HIAA, 5-hydroxyindoleacetic acid; SD, standard deviation. Similar letters indicate the groups being compared. **= *p* < 0.01.

### 3.4 Influence of disease-modifying therapy (DMT) on tryptophan, serotonin, and 5-HIAA serum levels in multiple sclerosis patients

The impact of DMT on TRP, 5-HT, and 5-HIAA levels in MS patients is not consistently demonstrated in direct longitudinal studies. Instead, DMT primarily exerts its effects by modulating neuroinflammatory pathways that influence TRP availability and the synthesis of 5-HT and 5-HIAA. Our statistical analysis shows a significant difference, H(6) = 2,169, *p* < 0.0014. Circulating levels of TRP, 5-HT, and 5-HIAA in MMS and WMS patients treated with rituximab, natalizumab, dimethyl fumarate, and fingolimod (Table 4) did not differ. However, the use of IFN-beta1a in WMS resulted in a significant decrease in circulating 5-HT levels (pmol/mL) compared to HW (WMSIFN 157.0 ± 88.0 vs. HWWT 306.0 ± 170.0, *p* < 0.01). Although our statistical analysis did not show any differences between the WMS group receiving glatiramer acetate, their average circulating 5-HT levels are 50% lower than those of the control group (WMSGA 167.0 ± 156.8 vs. HWWT 306.0 ± 170.0, *p* < 0.05).

**Table 4:** Serum levels of Trp, 5-HT, and 5-HIAA in participants stratified by treatment.

Treatment duration (years)	Healthy men (HM) *n* = 29	Serum levels of neurotransmitters (mean ± SD)	Men with MS (MMS) *n* = 29	Serum levels of neurotransmitters (mean ± SD)	Healthy women (HW) *n* = 84	Serum levels of neurotransmitters (mean ± SD)	Women with MS (WMS) *n* = 84	Serum levels of neurotransmitters (mean ± SD)
Unmedicated	*n* = 29	Trp = 13,320.0 ± 3,959.0 5-HT = 256.2 ± 86.6 5-HIAA = 851.7 ± 298.3	*n* = 3	Trp = 12,187 ± 4,178.0 5-HT = 231.8 ± 68.2 5-HIAA = 864.4 ± 331.1	*n* = 84	Trp = 12,846.0 ± 6,449.0 5-HT = 306.0 ± 170.0^**a/*b^ 5-HIAA = 834.0 ± 416.0	*n* = 8	Trp = 13,015 ± 6,293.0 5-HT = 305.6 ± 170.1 5-HIAA = 834.0 ± 415.7
Rituximab			*n* = 9	Trp = 12,939.0 ± 2,542.0 5-HT = 314.5 ± 113.6 5-HIAA = 722.1 ± 130.6			*n* = 17	Trp = 11,836.0 ± 1,995.0 5-HT = 278.0 ± 161.0 5-HIAA = 687.0 ± 167.0
Natalizu-mab			*n* = 3	Trp = 11,460.0 ± 1,642.0 5-HT = 149.9 ± 23.2 5-HIAA = 794.7 ± 44.4			*n* = 7	Trp = 12,906.0 ± 2,515.0 5-HT = 273.0 ± 226.0 5-HIAA = 690.0 ± 167.0
IFN-beta1a			*n* = 5	Trp = 11,537.0 ± 1,390.0 5-HT = 259.8 ± 101.8 5-HIAA = 707.7 ± 176.6			*n* = 20	Trp = 12,108.0 ± 2,846.0 5-HT = 157.0 ± 88.0** ^a^ 5-HIAA = 744.0 ± 128.0
GA			*n* = 6	Trp = 14,633 ± 4,618.0 5-HT = 229.1 ± 339.5 5-HIAA = 686.7 ± 339.5			*n* = 10	Trp = 9,849.0 ± 2,280.0 5-HT = 167.0 ± 156.8* ^b^ 5-HIAA = 781.0 ± 135.0
Dimetil-fumarato			*n* = 2	Trp = 11,904 ± 3,945.0 5-HT = 413.1 ± 87.8 5-HIAA = 602.7 ± 192.5			*n* = 9	Trp = 11,134.0 ± 4,730.0 5-HT = 332.0 ± 149.0 5-HIAA = 708.0 ± 131.0
Fingolimod			*n* = 1	Trp = 11,292.0 5-HT = 215.5 5-HIAA = 774.8			*n* = 12	Trp = 12,764.0 ± 2,273.0 5-HT = 341.0 ± 169.0 5-HIAA = 927.0 ± 744.0
Other							*n* = 1	Trp = 14,240.3 5-HT = 9,619.2 5-HIAA = 775.3

Trp, tryptophan; 5-HT, serotonin; 5-HIAA, 5-hydroxyindoleacetic acid; GA, Glatiramer acetate; SD, standard deviation. Similar letters indicate the groups being compared. *= *p* < 0.05; **= *p* < 0.01.

## 4 Discussion

The pathophysiology of MS is closely linked to disruptions in monoaminergic systems, as evidenced by altered tryptophan (Trp) metabolism and reduced levels of serotonin (5-HT) and its metabolite, 5-hydroxyindoleacetic acid (5-HIAA), in cerebrospinal fluid (CSF) (Markianos et al., 2013; Pashaei et al., 2022; San Hernandez et al., 2020). In addition to the disruption of the metabolic ratios of 5-HIAA/5-HT and melatonin/5-HT observed in the plasma of MS patients before treatment (Ricci et al., 2025). Our serum findings show a similar pattern, although the differences were not statistically significant. MMS patients had lower mean serum levels of Trp, 5-HT, and 5-HIAA than HM. Among WMS patients, Trp and 5-HIAA levels were also similarly reduced compared to HW.

Trp, the precursor to 5-HT, undergoes extensive peripheral catabolism, with about 95% metabolized via the kynurenine pathway (Cervenka et al., 2017). This pathway is stimulated by pro-inflammatory cytokines, particularly IFN-γ, through the activation of the inducible enzyme indoleamine 2,3-dioxygenase (IDO) (Pashaei et al., 2022; Cervenka et al., 2017; Gabela et al., 2025). Chronic inflammation in MS promotes the kynurenine pathway, reducing Trp availability for 5-HT synthesis and resulting in lower circulating 5-HT levels in serum, platelets, and the CNS. This serotonergic deficiency is implicated in the high prevalence of psychiatric comorbidities, especially depression, among MS patients (San Hernandez et al., 2020; Dolcetti et al., 2025).

Assessment of 5-HT levels reveals notable clinical associations with patient physical status. Individuals with lower body mass index (BMI) tend to exhibit significantly reduced CSF levels of 5-HIAA, a pattern also observed in secondary progressive MS (SPMS) (Markianos et al., 2013). Our findings confirm the relationship between low BMI and low circulating levels of 5-HT and 5-HIAA in WMS patients. Those with a BMI below 18 showed a significant reduction in serum 5-HT compared with HW (WMS: 164.2 ± 117.8 vs. HW: 305.6 ± 170.1; *p* < 0.05), while 5-HIAA levels present in WMS patients showed a downward trend without statistical significance (WMS: 515 ± 173.0 vs. HW: 778 ± 221.4). This coexistence of low BMI and diminished 5-HIAA may be associated with decreased homovanillic acid (a dopamine metabolite), reflecting dysfunction in central regulatory circuits that govern food reward and motivation (Markianos et al., 2013). Beyond this metabolic link, diminished serum and platelet 5-HT levels in MS patients are thought to result from reduced serotonin transporter (SERT) availability and increased diversion of Trp toward kynurenine pathway metabolism, through IDO activation (San Hernandez et al., 2020; Anderson and Rodriguez, 2015).

MS is a chronic, inflammatory, neurodegenerative disorder with a prolonged course—often spanning 30–40 years—characterized by accumulating neurological disability and frequent progression from relapsing–remitting (RRMS) to secondary progressive forms (SPMS), affecting about 80% of patients within 20–25 years after onset (S.F.M.S.E.T. University of California et al., 2016; Melnikov et al., 2022). This clinical trajectory is accompanied by significant disruptions in monoamine metabolism, particularly involving Trp, 5-HT, and 5-HIAA (San Hernandez et al., 2020). Chronic neuroinflammation in MS triggers IDO activation, diverting Trp metabolism away from 5-HT synthesis and resulting in consistently low 5-HT levels in serum, platelets, and the CNS (Anderson and Rodriguez, 2015; Tauil et al., 2020). This serotonergic deficit, reflected by reduced CSF 5-HIAA in progressive disease phenotypes, is strongly associated with the high prevalence of psychiatric comorbidities such as depression and cognitive fatigue in MS (San Hernandez et al., 2020; Dolcetti et al., 2025; Tarasiuk et al., 2021). In our cohort, only WMS patients with treatment duration greater than 4 years showed significantly lower serum 5-HT than HW (WMS: 210 ± 146.0 vs. HW: 306 ± 170.0), with 5-HIAA levels also trending lower, although not reaching significance.

Our analysis of treatment effects showed that serum levels of Trp, 5-HT, and 5-HIAA remained unchanged in patients receiving rituximab, natalizumab, dimethyl fumarate, or fingolimod. In contrast, a significant decrease in 5-HT levels was observed in WMS treated with IFN-β. WMS treated with glatiramer acetate (GA) showed a 50% decrease in levels compared to HW.

IFN-β1a is a widely used first-line disease-modifying therapy (DMT) approved for relapsing forms of MS (e.g., IFN-1a, Peg-IFN-1a) (Simoens, 2022; Tauil et al., 2020). Mechanistically, IFN-β decreases antigen presentation, suppresses Th1 proliferation, increases IL-10 production, modulates co-stimulatory molecules, and restores T regulatory (Treg) cell activity by reducing D1-like dopamine receptor expression (Simoens, 2022; Pashaei et al., 2022). Notably, IFN-β has been shown to induce kynurenine pathway activation in human macrophages (Tauil et al., 2020). The association between IFN-β therapy and depression in MS patients suggests that the drug—or the underlying inflammation prompting its use—may exacerbate neurochemical imbalances (Bonaccorso et al., 2009). In our study, WMS patients treated with IFN-β1a had significantly lower serum 5-HT levels than HW (WMS: 157.0 ± 88.0 vs. HW: 306 ± 170.0; *p* ≤ 0.01).

GA is a heterogeneous synthetic peptide composed of four amino acids (L-glutamic acid, L-lysine, L-alanine, and L-tyrosine) (Campos-Garcia et al., 2017). As a first-line immunomodulatory therapy for RRMS, GA aims to slow disability progression and reduce relapse frequency (Jakimovski et al., 2024; Campos-Garcia et al., 2017). Its action is primarily immunological, shifting T cell responses from a pro-inflammatory Th1 phenotype to an anti-inflammatory Th2 phenotype (San Hernandez et al., 2020; Avila et al., 2019). This mechanism suggests that GA could mitigate chronic inflammation-driven Trp depletion and subsequent 5-HT deficiency. However, studies examining changes in Trp and 5-HIAA after GA initiation report inconsistent results (Anderson and Rodriguez, 2015; Tauil et al., 2020). In our cohort, WMS who received GA had lower serum 5-HT levels than HW (WMS: 167.0 ± 156.8 vs. HW: 306 ± 170.0), which contrasts with prior reports suggesting GA treatment ameliorates depression, anxiety, and cognitive deficits, potentially by improving 5-HT status (Salihu et al., 2021).

The data from this study may be better interpreted when its limitations are considered. Reliance on peripheral serum measures of 5-HT and 5-HIAA provides only an indirect indication of central serotonergic activity, especially without cerebrospinal fluid data or neuroimaging correlates. The limited sample size reduces statistical power and restricts the generalizability of subgroup comparisons. The cross-sectional design precludes causal inference, so the findings should be regarded as associative rather than mechanistic. The absence of clinical-psychiatric follow-up underscores the need for future longitudinal studies.

## 5 Conclusion

Multiple sclerosis (MS) shows pronounced sexual dimorphism that influences under certain condition disease incidence, clinical progression, inflammatory responses, and molecular pathways, which necessitate sex-stratified therapeutic and prognostic strategies. Previous studies from our group have documented sex-dependent differences in epigenetic regulation (Reyes-Mata et al., 2023), cytokine production (Guerrero-Garcia Jde et al., 2016), and humoral responses (Avila et al., 2019) in MS cohorts, depending on gender. This cross-sectional study builds on previous findings by showing that peripheral 5-HT reductions in RRMS are influenced not only by sex but also by clinical factors such as low body mass index (BMI < 18 kg/m^2^), longer treatment duration (>4 years), and specific disease-modifying therapies (DMTs), with female patients showing decreased 5-HT levels. These sex-specific, BMI-, duration-, and DMT-associated serotonergic deficits highlight chronic inflammation-driven activation of the kynurenine pathway via indoleamine 2,3-dioxygenase (IDO), which diverts tryptophan from serotonin synthesis and contributes to psychiatric comorbidities such as depression (Cervenka et al., 2017; Dolcetti et al., 2025).

Although our findings need to be replicated in other populations affected by MS, nevertheless, this novel integration of peripheral biomarkers with clinical variables suggest a probable clinical utility: routine psychiatric and nutritional assessments in women with MS who have low BMI, prolonged disease duration (>4 years), or are receiving IFN-β1a therapy could improve quality of life by preempting neuropsychiatric complications, pending replication in diverse cohorts. These findings build on recent evidence linking serotonin dysregulation to MS progression through adenosine deaminase (ADA) inhibition, further supporting the relevance of serotonergic pathways as modifiable targets in sex-dimorphic MS management. In addition, we propose that psychiatric and clinical evaluation of women with MS, low BMI, disease duration of more than 4 years, and treatment with IFN beta-1a may improve the quality of life for patients and their families by preventing further complications.

## Data Availability

The raw data supporting the conclusions of this article will be made available by the authors, without undue reservation.

## In memoriam

This manuscript is dedicated to Fernando Pavón Soriano (1950–2025), in heartfelt gratitude for his unwavering affection, steadfast support, and the boundless love he shared with us throughout his life. His memory continues to inspire and guide us.
